# The Association between Iron-deficiency Anemia and Adverse Pregnancy Outcomes: A Retrospective Report from Pakistan

**DOI:** 10.7759/cureus.5854

**Published:** 2019-10-07

**Authors:** Tuba Mahmood, Atique Ur Rehman, Gantuya Tserenpil, Faiza Siddiqui, Mehak Ahmed, Fatima Siraj, Besham Kumar

**Affiliations:** 1 Cardiology, Quaid-e-Azam Medical College, Bahawalpur, PAK; 2 Internal Medicine, Quaid-e-Azam Medical College, Bahawalpur, PAK; 3 Internal Medicine, Mongolian National University of Medical Sciences, Ulaanbaatar, MNG; 4 Anatomy, Liaquat College of Medicine and Dentistry, Karachi, PAK; 5 Medicine, Liaquat College of Medicine and Dentistry, Karachi, PAK; 6 Miscellaneous, Liaquat College of Medicine and Dentistry, Karachi, PAK; 7 Internal Medicine, Jinnah Postgraduate Medical Center, Karachi, PAK

**Keywords:** antenatal anemia, maternal anemia, adverse pregnancy outcomes, neonatal outcomes, anemia in pregnancy

## Abstract

Background

Anemia is one of the most common conditions that affect pregnancies, with dietary iron deficiency being its most common cause. Maternal anemia has been associated with increased risks of both maternal and neonatal adverse outcomes. This study aimed to analyze the maternal and neonatal outcomes in women with third-trimester anemia.

Methods

This was a retrospective report from a Pakistani public hospital. It included data records of the childbirths in the hospital, with at least one record that documented the hemoglobin (Hb) level in women in the first or second trimester and one in the third trimester. The duration of the study was from January 1, 2019 to June 30, 2019. Women with Hb level of <10mg/dL in the third trimester were categorized as anemic, and those with Hb level of >10mg/dL were categorized as non-anemic. Pregnancy outcomes were assessed for both mothers and babies. All data were processed through SPSS version 21.0 for Windows (IBM Corp., Armonk, NY).

Results

The study evaluated 235 (37.8%) anemic and 387 (62.2%) non-anemic women. Adverse maternal outcomes were compared between the two groups. In anemic women, gestational hypertension (56% vs. 27%; p: <0.0001), preeclampsia (65% vs. 25%; p: <0.0001), antepartum hemorrhage (32% vs. 19%; p: =0.0001), postpartum hemorrhage (79% vs. 28%; p: <0.0001), transfusions (94% vs. 5%; p: <0.0001), prolonged/obstructed labor (49% vs. 20%; p: <0.000), urgent induction of labor (24% vs. 2%; p: <0.0001), and urgent caesarean section (CS) (45% vs. 29%; p: 0.0001) were significantly more common as compared to non-anemic women. Adverse neonatal outcomes such as low birth weight (LBW) (59% vs. 29%; p: <0.0001), small-for-gestational-age (SGA) (73% vs. 23%; p: <0.0001), preterm delivery (39% vs. 15%; p: <0.0001), stillbirth (8% vs. 3%; p: 0.01), and early neonatal death (9% vs. 2%; p: 0.000) were associated more with anemia. There was no report of maternal mortality in either group.

Conclusion:

Anemia in the third trimester of pregnancy is associated with adverse maternal and neonatal outcomes including neonatal death. Efforts are required to ensure adequate maternal nutritional status in order to prevent poor outcomes.

## Introduction

Anemia is among the most common conditions that affect pregnancies. Anemia in pregnancy has a varied incidence and etiology depending on the geographic location [1]. The incidence of anemia has been as high as 35-75% in developing countries compared with only 19% in developed countries [2].

According to guidelines published in the UK in collaboration with the British Society for Haematology (BSH), Obstetric Haematology Group (BSH OHG), and British Committee for Standards in Haematology (BCSH), anemia in pregnancy is defined as the condition where serum hemoglobin (Hb) level is <11.0 mg/dl in the first trimester, <10.5 mg/dl in the second and third trimesters, and <10.0 mg/dl in the postpartum period [3].

There are several pathologies that cause anemia in pregnancy, including acute and chronic infections, disorders of hemoglobin synthesis, and nutritional deficiencies such as deficiency of vitamin B12, folic acid, and iron. Dietary iron deficiency is the most common cause of anemia in pregnancy [4]. Irrespective of the etiology, maternal anemia has been associated with increased risks of both maternal and neonatal adverse outcomes. In a study conducted in Israel, maternal anemia was found to increase the risk of cesarean sections (CS) and the need for blood transfusions. The same study also observed that the neonates of anemic women had an increased risk of a low APGAR score [5]. Other adverse outcomes include preterm delivery [6], small-for-gestational-age (SGA) (weight below the 10th percentile for the gestational age) [7], postpartum hemorrhage (PPH), and preeclampsia [8,9]. Even though the trends in Pakistani neonates have been studied from several angles [10-12], little attention has been given to maternal outcomes [9]. Hence, this study was designed to analyze the maternal and neonatal outcomes in women with third-trimester anemia.

## Materials and methods

This was a review of a historical cohort of all patients delivering in the obstetrics and gynecology department of a tertiary care hospital in Pakistan from January 1, 2019 to June 30, 2019. This study was approved by the institutional review board. Only those data records that had at least one record of hemoglobin (Hb) level in the first or second trimester and one in the third trimester were included.

A semi-structured proforma was constructed to record maternal and neonatal information. Patients were divided into two groups based on their Hb level in the third trimester. Women with Hb level of <10mg/dL were categorized as anemic, and those with Hb level of >10mg/dL were categorized as non-anemic [3]. Demographic characteristics including age, body weight, height, smoking status, and parity were recorded. Clinical characteristics including the history of antenatal visits and outcomes of previous pregnancies, iron supplementation status, and Hb level in the first and/or second trimester of current pregnancy were recorded. Pregnancy outcomes were assessed for both the mothers and babies. Maternal outcomes included in the study were gestational hypertension, preeclampsia, eclampsia, antenatal hemorrhage (any bleeding from the genital tract at 24+0 weeks of gestation till before delivery), postpartum hemorrhage (>500 ml of blood loss during or within 24 hours of delivery), obstruction of labor (OL)/prolonged labor (PL), induction of labor (IOL), blood transfusions, emergency CS/laparotomy, and maternal mortality. Fetal outcomes included fetal distress, low birth weight (LBW) (less than 2,500 grams), SGA, prematurity (childbirth before 37+0 weeks of gestation), neonatal intensive care unit (NICU) admission, stillbirth, and early neonatal death.

Statistical analysis was completed through SPSS version 21.0 for Windows (IBM Corp., Armonk, NY). Categorical data were presented as frequencies and percentages. Continuous data were presented as mean and standard deviation (SD). A chi-square test was performed to analyze the correlation between categorical variables. A p-value of ≤0.05 was considered significant.

## Results

There were 622 records included in the analysis. The mean age of the women was 29.4 (±12.5) years. The mean BMI of the women was 29.3 (±6.4) kg/m^2^, and their mean parity was 5 (±3). There were 235 (37.8%) women who were anemic in their third trimester and 387 (62.2%) who were non-anemic. Their baseline characteristics were compared. It showed that anemic women were younger in age, less likely to have a normal body weight, more likely to be active as well as passive smokers, and more likely to be primiparous (Table 1). 

**Table 1:** Comparison of baseline demographic characteristics of the anemic and non-anemic study population

Baseline characteristics	Anemic (n = 235; 37.8%)	Non-anemic (n = 387; 62.2%)	P-value
Maternal age, years			
Less than 20	93 (39.5%)	102 (26.3%)	0.002
20-30	88 (37.4%)	172 (44.4%)	
More than 30	54 (23.0%)	113 (29.2%)	
BMI, kg/m^2^			
Underweight (<18.5 kg)	123 (52.3%)	89 (22.9%)	<0.0001
Normal (18.5-24.9 kg)	37 (15.7%)	193 (49.8%)	
Overweight (25-29.9 kg)	43 (18.3%)	78 (20.2%)	
Obese (>30 kg)	32 (13.6%)	27 (6.9%)	
Smoking status			
Active smoker	53 (22.5%)	64 (16.5%)	<0.0001
Passive smoker	201 (85.5%)	156 (40.3%)	
Ex-smoker	19 (8.1%)	54 (13.9%)	
Non-smoker	163 (69.4%)	269 (69.5%)	
Parity			
0	102 (43.4%)	95 (24.5%)	<0.0001
1-3	56 (23.8%)	126 (32.5%)	
4-6	14 (5.9%)	75 (19.4%)	
More than 7	63 (26.8%)	91 (23.5%)	

In the anemic group, 52% had been anemic since their first trimester and the percentage increased to 66% in the second trimester as compared to 30% in the first and 20% in the second trimester in the non-anemic group. The mean Hb level of the anemic group was 6.8 (±2.5) mg/dL in the first trimester, 7.3 (±1.8) mg/dL in the second, and 7.2 (±1.3) mg/dL in the third trimester. The mean Hb level of the non-anemic group was 8.1 (±2.7) mg/dL in the first trimester, 8.8 (±2.9) mg/dL in the second, and 8.9 (±1.9) mg/dL in the third trimester. A comparison of the clinical characteristics of both groups was performed, which showed that anemic women had significantly lower Hb values in first and second trimesters, poor compliance to antenatal visits, lower frequency of iron supplementation, and more frequent treatment for iron deficiency (Table 2).

**Table 2:** Comparison of clinical characteristics of the anemic and non-anemic study population

Clinical characteristics	Anemic (n = 235; 37.8%)	Non-anemic (n = 387; 62.2%)	P-value
Hemoglobin level in the first trimester, g/dL			
Less than 8	123 (52.3%)	115 (29.7%)	<0.0001
8-10	82 (34.8%)	145 (37.5%)	
More than 10	9 (3.8%)	100 (25.8%)	
Missing	21 (8.9%)	27 (6.9%)	
Hemoglobin level in the second trimester, g/dL			
Less than 8	155 (65.9%)	78 (20.2%)	Not applicable
8-10	44 (18.7%)	203 (52.4%)	
More than 10	0 (0%)	99 (25.5%)	
Missing	36 (15.3%)	7 (1.8%)	
Iron supplements taken			
In current pregnancy	68 (28.9%)	128 (33.1%)	<0.0001
In previous pregnancies	103 (43.8%)	235 (60.7%)	
Never during pregnancy	64 (27.2%)	24 (6.2%)	
Intravenous iron replacement			
In current pregnancy	104 (44.3%)	21 (5.4%)	<0.0001
In previous pregnancies	115 (48.9%)	66 (17.1%)	
Never during pregnancy	29 (12.3%)	300 (77.5%)	
Antenatal visits in previous pregnancies (not valid for primigravida)			
None	53/133 (39.8%)	33/292 (11.3%)	<0.0001
One only	58/133 (43.6%)	101/292 (34.5%)	
More than one	22/133 (16.5%)	158/292 (54.1%)	
Outcome of previous pregnancies (not valid for primigravida)			
Alive and healthy	103/133 (77.4%)	279/292 (95.5%)	<0.0001
Abortion/fetal death/stillbirth	21/133 (15.8%)	10/292 (3.4%)	
Neonatal/early-childhood death	9/133 (6.8%)	3/292 (1.0%)	

Maternal and neonatal outcomes were compared. In the anemic women group, pregnancy-related complications including gestational hypertension, preeclampsia, eclampsia, antepartum, and postpartum hemorrhage were more common. There was a higher frequency of obstructed/prolonged labor and fetal distress leading to induction and/or emergency CS/laparotomy in the anemic group. The neonatal outcome was also poor in the anemic group. There were no maternal mortalities in our study. There were 29 (4.7%) neonatal deaths, out of which 21 (3.4%) pertained to anemic mothers (Table 3).

**Table 3:** Comparison of maternal and neonatal outcomes of the anemic and non-anemic study population NICU: Neonatal intensive care unit

Pregnancy outcomes	Anemic (n = 235; 37.8%)	Non-anemic (n = 387; 62.2%)	P-value
Gestational hypertension	131 (55.7%)	103 (26.6%)	<0.000
Preeclampsia	153 (65.1%)	98 (25.3%)	<0.000
Eclampsia	5 (2.1%)	0 (0%)	Not applicable
Antepartum hemorrhage	76 (32.3%)	73 (18.8%)	0.0001
Postpartum hemorrhage (>500 ml)	185 (78.7%)	107 (27.6%)	<0.000
Major obstetric hemorrhage (>2,000 ml)	58 (24.7%)	12 (3.1%)	<0.000
Need for blood transfusion during/within 24 hours of delivery	221 (94.0%)	19 (4.9%)	<0.000
Prolonged/obstructed labor	115 (48.9%)	76 (19.6%)	<0.000
Need of urgent laparotomy due to uncontrollable bleeding	12 (5.1%)	0 (0%)	Not applicable
Need of urgent induction of labor due to maternal/fetal distress	56 (23.8%)	8 (2.1%)	<0.000
Need of urgent caesarean section due to fetal distress/prolonged labor/failure of induction	105 (44.6%)	113 (29.2%)	0.0001
Low birth weight (<2,500 g)	138 (58.7%)	114 (29.5%)	<0.000
Very low birth weight (<1,500 g)	78 (33.2%)	13 (3.3%)	<0.000
Small-for-gestational-age	173 (73.6%)	89 (22.9%)	<0.000
Preterm delivery	93 (39.5%)	58 (14.9%)	<0.000
Stillbirth	18 (7.6%)	12 (3.1%)	0.01
Early neonatal death (within 24 hours)	21 (8.9%)	8 (2.1%)	0.000
NICU admission	135 (57.4%)	94 (24.3%)	<0.000

## Discussion

Maternal anemia is a common problem encountered by gynecologists and obstetricians worldwide, especially in developing countries. In this study, we found an association between anemia in pregnancy and adverse maternal and neonatal outcomes. While this study throws light on the issue in a developing country, its results cannot be generalized as it was based on cases at a single facility. It lacks demographic diversity, and there is a high probability of missing/incomplete data due to its retrospective nature.

Other studies have shown similar results to ours. Nair et al. studied a large retrospective cohort from India in which 35% of pregnant women had moderate-to-severe anemia. This study reported that anemic women had a nine times higher risk of PPH. For anemic women who underwent an IOL, the risk increased 17-fold; and for anemic women with infections, it increased 19-fold. Adverse neonatal outcomes associated with anemia were LBW, SGA, and perinatal death [13]. SGA, LBW, and preterm delivery have been reported in other studies from India as well [9,14]. Parks et al. also reported a significant association between severe anemia and PPH and neonatal mortality, but no connection between severe anemia and maternal mortality was observed [9].

The trends from other developing countries such as Sudan, Tanzania, and Bangladesh have not been very different either [13-20]. Neonatal adverse outcomes associated with maternal anemia in these countries include low placental weight, LBW/very LBW, poor APGAR score, SGA, birth asphyxia, fetal anemia, stillbirth, and preterm delivery [13-20]. Maternal adverse outcomes reported in the literature include preeclampsia, PPH, CS delivery, and infections [19,9,21].

Although the incidence of nutritional anemia in pregnancy is low and is in a further decline in developed countries, it is still associated with poor maternal outcomes [22]. In a Scottish retrospective cohort, maternal anemia reportedly increased the risk of antepartum hemorrhage, severe obstetric hemorrhage, the need for blood transfusion, postpartum infection, and maternal death [22]. Among Finnish multiparous women, antenatal anemia was associated with preterm delivery, SGA, and NICU admission [23]. Demuth et al. reported that less than half of the German women diagnosed with iron-deficiency anemia were taking a therapeutic dose of supplemental iron during their pregnancy [24].

Currently, the management of labor and delivery in women with moderate-to-severe anemia is not governed by any standard guidelines [25,26]. The frequency of anemia in the last trimester is on the higher side (38%) as reported in this study. It carries a high risk of poor maternal and fetal outcomes. There is an urgent need to find more evidence and design high-quality protocols on adequate management of women with moderate-to-severe anemia during labor, delivery, and the postpartum period. We believe this study has highlighted the incidence and consequences of antenatal anemia among Pakistani women. There is evidently a pressing need to address maternal nutrition and the general health of women of childbearing age. The need for more interventional studies that can include larger samples at an epidemiological level and anemias of various etiologies cannot be overstated. Studies must be undertaken to assess the value of treating anemia as an independent risk factor in predicting the outcome of pregnancy. There should be an increased focus on improving awareness about the positive impact of adequate micronutrient replenishment on the health of the mother as well as the newborn.

## Conclusions

Maternal anemia is associated with adverse maternal and neonatal outcomes. It is important to identify women at risk and ensure that sufficient and timely care is provided. Awareness campaigns must be conducted to educate women about the need to take care of their health and well-being during pregnancy in order for them to have healthy babies. The focus should be placed on proper and adequate maternal nutrition, and women must be urged to seek antenatal care on a regular basis. Appropriate antenatal care will help reduce adverse preventable outcomes such as third-trimester anemia. Awareness campaigns should not be confined to pregnant women, and all women of childbearing age must be educated about the importance of adequate maternal nutrition.
